# Anion-controlled dimer distance induced unique solid-state fluorescence of cyano substituted styrene pyridinium

**DOI:** 10.1038/srep37609

**Published:** 2016-11-21

**Authors:** Gaobin Zhang, Xuanjun Zhang, Lin Kong, Shichao Wang, Yupeng Tian, Xutang Tao, Jiaxiang Yang

**Affiliations:** 1 College of Chemistry & Chemical Engineering, Key Laboratory of Functional Inorganic Materials of Anhui Province, Anhui University, Hefei 230601, P. R. China; 2 Faculty of Health Sciences, University of Macau, Taipa, Macau SAR, P. R. China; 3 State Key Laboratory of Crystal Materials, Shandong University, Jinan 502100, P. R. China

## Abstract

Molecular packing arrangements play a key role in dominating the photophysical properties of luminophores in aggregated state but fine control of the molecular packing is a great challenge. This article describes a unique cyano substituted styrene pyridinium with interesting solid-state fluorescence that can be finely tuned by simple change of counteranions. The dilute solutions of the organic salts (**PyCl**, **PyNO**_**3**_, **PyOTs** and **PyPh**_**4**_**B**) exhibit very weak fluorescence. The crystals of the organic salts (**PyCl**, **PyNO**_**3**_, and **PyOTs**) show much enhanced fluorescence compared with their dilute solutions. It is interesting that the emissions changed from bluish-green to deep-blue and fluorescence quantum yields increase from 2.5% to 13.1% with the increasing of steric hindrance of the anions from chloridion, nitrate, to p-toluenesulfonate. Crystal and DFT studies reveal that the enhanced fluorescence is ascribed to the formation of dimers and bigger anions induce larger molecular separation in dimers. Tetraphenylboron anion with very large steric hindrance impedes the formation of dimers and thus results in non-fluorescent salt (**PyPh**_**4**_**B**). Meanwhile, this unique dimeric packing endows the crystal of **PyNO**_**3**_ with anisotropic fluorescence.

Conjugated organic luminophors have wide applications in organic light-emitting diode (OLEDs)^1^,^2^,^3^,^4^,^5^, smart materials^6^,^7^,^8^,^9^,^10^ and sensors^11^,^12^,^13^,^14^,^15^. Understanding the structure-property relationship is very important to guide the design of correct molecules for their providential applications^16^,^17^,^18^,^19^,^20^,^21^,^22^,^23^. Artful molecular design through varying molecular backbone substituent groups and effective π-conjugation lengths or impacting the intra/intermolecular interactions to achieve high emission in the solid state is the most common method^24^,^25^,^26^,^27^,^28^. In contrast the notorious aggregation-caused quenching (ACQ), branchy and twisted aggregation-induced emission (AIE) and aggregation-induced emission enhancement (AIEE) fluorogens are developed to tune the intermolecular stacking for efficient emissions in the solid state^29^,^30^,^31^,^32^. Alternative ways to regulate multifunctional materials are allomorphism and mischcrystal^33^,^34^,^35^. This strategy do not involves tedious organic synthesis. However, the requirements for the molecular structures are stringent and the distinct properties commonly disappear when the crystals are damaged.

Organic salts have advantages for the design of solid-state emitters because the photophysical properties could be tuned by both their cation and anion constituents^36^,^37^,^38^,^39^,^40^,^41^,^42^,^43^. The cationic fluorogens are usually designed with complex and flexible structures to against the strong dipole-dipole interactions, which open the non-radiation decay channels and thus quench the solid-state luminescence. The changes of the anions would also greatly influence the emission in aggregation of organic salts because the strength of electrostatic attraction between cationic and anionic species and steric hindrance may deeply affect the molecular packing in aggregation^44^,^45^,^46^,^47^,^48^,^49^,^50^,^51^. Bhattacharya, *et al*. reported a phenylenedivinylene bis-N-octyl pyridinium salt^52^. The salt effect profoundly influenced the order of aggregation of the gelator molecules to form a novel chromophore assembly leading to an aggregation-induced switch of the emission colors. Tang’s group found that effect of the counterion could change the emission behavior of (TPE)-functionalized benzothiazolium salts from ACQ to AIE^53^. While a number of literatures focus on the effect of the counterion on the photophysical characteristics of organic salts, the definite and visual evidences of the structure-property relationship are seldom reported as far as we know.

In this work, a series of cyano substituted styrene pyridinium salts (Fig. 1) were prepared, which exhibited unique anion-controlled solid-state fluorescence. The compound **PyCl** was synthesized in high yield using pyridylaldehyde and phenylacetonitrile by Knoevenagel reaction followed by reaction with benzylchloride. **PyNO**_**3**_, **PyOTs**, and **PyPh**_**4**_**B** were obtained by exchange of the anions in methanol using silver nitrate, silver p-toluenesulfonate and tetraphenylborate, respectively. It is interesting that these four organic salts exhibit remarkable differences in solid-state fluorescence.

## Results and Discussion

**Figure 2:**
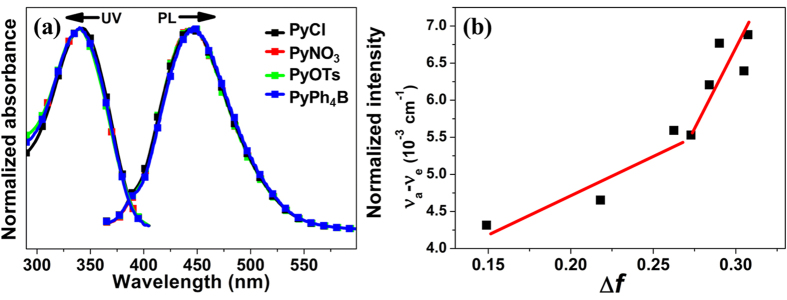
**(a)** Absorptions and fluorescence spectra of the four compounds in acetonitrile (10 μM). **(b)** Linear correlation of the Stokes’ shift with the solvents orientation polarization for **PyCl**.

To investigate the effect of counterion, the photophysical properties of **PyCl**, **PyNO**_**3**_, **PyOTs** and **PyPh**_**4**_**B** in various dilute organic solvents with different polarities are firstly studied. As depicted in Fig. 2, the absorption and fluorescence spectra of the all compounds in dilute solutions show similar profiles. The main peaks of their absorption spectra locate at about 345 nm, which have very little changes in different organic solvents (Figure S1). Meanwhile, evidence solvatochromic effects were observed (Figure S2). With the increasing of the polarity of solvents, the emission peaks showed red-shifted from about 420 nm to about 445 nm, which indicated a strong intramolecular charge transfer (ICT). The Lippert–Mataga relation was used to better understand the solvent-polarity effect. Interestingly, the plot of the orientation polarizability (Δ*f*) versus the Stokes’ shift of the compounds shows two sets of linearity indicative of two different excited states (Fig. 2b and Figures S3–5). The larger slope in the strong polarity solvents shows a typical character of CT state^54^. All above, the similar Lippert-Mataga relation, absorption and fluorescence of **PyCl**, **PyNO**_**3**_, **PyOTs** and **PyPh**_**4**_**B** in various dilute organic solvents in which molecules are monomers indicate that the anions have no effect on the photophysical properties of the cationic fluorogen and the interactions between cations and anions could not change the decay channel of cationic fluorogen monomers.

The strengthened interactions between anions and cations in the solid state have deep effect on the intra/intermolecular packing arrangements, which could be reflected on the photophysical properties. The pyridinium salt is easily dissolved in the polar solvents such as MeOH, EtOH, acetonitrile and DMF, but insoluble in toluene, benzene and THF. Solvent/non-solvent experiments were carried out to investigate the aggregation behaviors by controlling the acetonitrile/toluene ratios. The absorption (Figure S6) and fluorescence (Figure S7) spectra of **PyCl**, **PyNO**_**3**_, **PyOTs** and **PyPh**_**4**_**B** were found to be dependent on the toluene fraction. All of the compounds in acetonitrile is weakly emissive because of the twisted intermolecular charge transfer (TICT). When the non-solvent toluene is added, the fluorescence spectra of **PyCl**, **PyNO**_**3**_ and **PyOTs** present almost the same tendency. With the increasing of the toluene fractions (*f*_T_), the maximal emission gradually enhanced. It is noted that the maximal intensity of **PyCl**, **PyNO**_**3**_ and **PyOTs** is increased with the increasing of the steric of the anions. These results reveal that **PyCl**, **PyNO**_**3**_ and **PyOTs** are AIEE active. Very interestingly, **PyPh**_**4**_**B** with the largest anion shows weak emission both in dilute solution and aggregate without AIEE effect. The fluorescence intensity was gradually decreased with the increasing of *f*_T_ in acetonitrile/toluene mixture. The results demonstrate that the aggregation of **PyPh**_**4**_**B** is difference from those of **PyCl**, **PyNO**_**3**_ and **PyOTs** and the molecular packing arrangements are anion-dependent.

**Figure 3:**
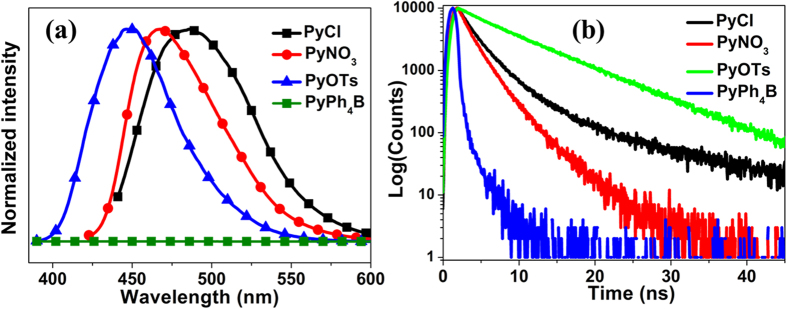
**(a)** Fluorescence spectra and **(b)** lifetimes of the compounds in the solid state.

The solid-state fluorescence was also investigated using microcrystals. As shown in Fig. 3, the emission wavelengths of **PyCl**, **PyNO**_**3**_ and **PyOTs** are at 486, 467 and 448 nm, respectively, which indicate that the fluorescence are blue-shifted with the increasing of steric of anions. Fluorescence quantum yields (Ф_F_) of **PyCl**, **PyNO**_**3**_, **PyOTs** and **PyPh**_**4**_**B** in the solid state were determined by a calibrated integrating sphere. The Ф_F_ values of these compounds were quite different (Table 1). **PyOTs** with the second larger anions exhibits the highest Ф_F_ value of 13.1%. However, **PyPh**_**4**_**B** with the largest anion of tetraphenyl borate is nearly non-emissive in the solid state (Ф_F _< 1%). The Ф_F_ value of **PyNO**_**3**_ is 10.4%, which is higher than that of **PyCl** (2.5%).

Crystallographic studies are important to understand the roles of anions on the packing arrangement and the fluorescence mechanism. Fortunately, single crystals suitable for X-ray diffraction were obtained for all of the four salts. The detailed crystal and structure refinement data are given in Table S1. The packing arrangements of **PyCl**, **PyNO**_**3**_, **PyOTs** are “bricks” with the anions inserted in the space between cationic fluorogens (Figures S8–10). However, the molecular packing of **PyPh**_**4**_**B** is different from the others (Figure S11). The molecular packing is stabilized by multiple interactions between the cations and anions. The steric effects of anions significantly affect the molecular planarity and packing of the cations. The monatomic chloridion in **PyCl** formed three H-bond on the same plane leading to better planarity of the cation, in which the pyridyl, vinyl and phenyl are almost co-planar. However, the nitrate anion is in a polyhedron conformation. Different oxygen atoms of nitrate anion form three H-bonds with the H atoms located at the pyridyl, vinyl, and phenyl, respectively, which results in a twisted structure. In the crystal of **PyOTs** with larger anion p-toluenesulfonate, the cations are also non-planar (Figures S12–13).

**Figure 4:**
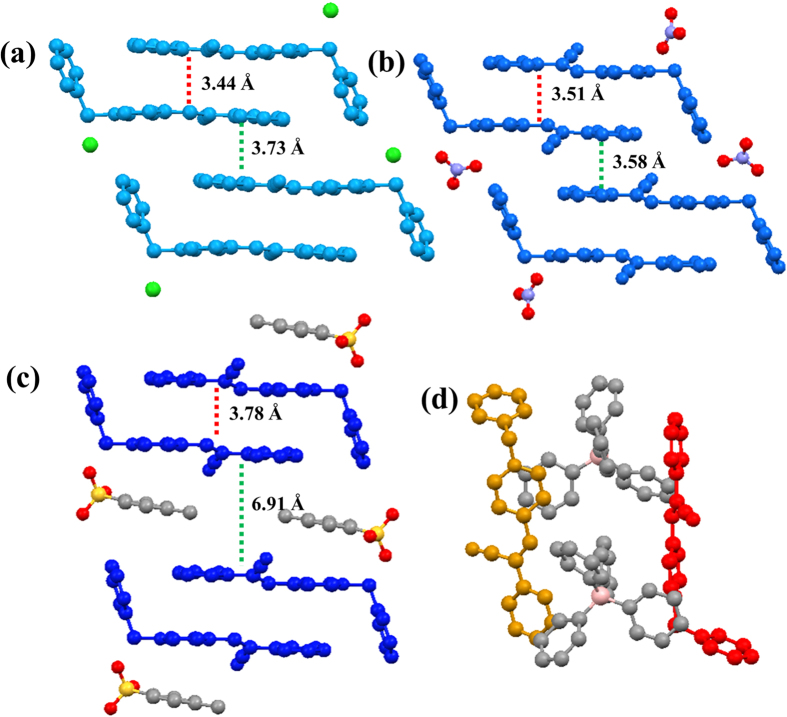
The molecular packing arrangements in crystals **PyCl (a)**, **PyNO**_**3**_ **(b)**, **PyOTs (c)** and **PyPh**_**4**_**B (d)**.

The D-π-A type molecules have the tendency to form H-aggregate by the coulombic force^55^,^56^,^57^,^58^. The **PyCl**, **PyNO**_**3**_ and **PyOTs** formed H-aggregate dimers by the face-to-face stacking of the electron-deficient pyridinium cation and the relative electron-rich phenyl. Moreover, the degree of π-π stacking of **PyCl**, **PyNO**_**3**_ and **PyOTs** dimers are controlled by the multiple interactions between cations and anions through conducting the distance of the paired molecules (Fig. 4). In the crystal of **PyCl**, as a result of the planarity structure of cation and small size of anion, the distance between two monomers in a dimeric pair is short (3.44 Å), which indicates the strong π-π stacking between the paired molecules. Affected by the steric hindrance of nitrate anions, the intradimeric distance of **PyNO**_**3**_ was 3.51 Å, which was slightly longer than that in **PyCl**. Distance between the paired molecules of in **PyOTs** was the longest (3.78 Å), which indicates a weak π-π stacking between the paired monomers. Besides, the C-H_+++_π and H-bond interactions between the anions and cations increased the overlap of the paired molecules. Thus, the steric hindrance of anions tune the distance between two dimeric molecules that result in the different degree of intradimeric interactions.

On the other hand, the distance between neighboring dimers was dominated by both the ionic interaction and steric hindrance. The dimer to dimer distance in **PyNO**_**3**_ crystal was 3.58 Å, which was shorter than that of 3.73 Å in **PyCl**. We found that dimers in **PyNO**_**3**_ were drawn up by several H-bonds between nitrate anion and two neighboring dimers. While, there is no direct interaction between chloridion and neighboring dimers in **PyCl** (Figures S14–15). In **PyOTs**, the p-toluenesulfonate anions had H-bond interaction between two neighboring dimers. However, the steric effect of p-toluenesulfonate anions was dominated here, which was inserted in the space between dimers leading to the larger separation of 6.91 Å between dimers (Figure S16). The packing arrangements of cations in **PyPh**_**4**_**B**, interestingly, are totally different to the other analogues. The large steric hindrance of tetraphenylboron anion impedes the electrostatic interactions of two cation fluorogens (Figure S17). The cations are “diluted” by the tetraphenylboron anions and present as single monomers in the crystal. The dimers are not formed and the electrostatic interactions take place of the intra/interdimeric interactions.

The solid-state fluorescence is ascribed to the decay of the dimeric excimers. These unique AIEEgens are different from other common ones with the mechanism of restriction of intramolecular rotations or intramolecular planarization. Steric hindrance of anions and the interactions between the anions and cations have dominating roles in the intra/interdimeric packing arrangements. Tetraphenylboron anions impede the formation of dimeric aggregates and result in very weak fluorescence of **PyPh**_**4**_**B** in the solid state (Ф_F_ < 0.01). On the other hand, **PyCl**, **PyNO**_**3**_, and **PyOTs** form dimeric aggregates possess AIEE properties with strong emission in the solid state. The chloridion with small steric makes the **PyCl** easily form dimers. Its small intradimeric distance increases the π-conjugate overlap of the monomers, which induces the excimers emission. However, the strong π-π stacking opens the non-radiation channels, which results in relatively weak emission in the solid state. With the increasing of the steric hindrance of the anions, the intradimeric distance is increased. The decreased intradimeric π-π stacking leads to enhanced fluorescence of **PyNO**_**3**_ and **PyOTs** with blue-shifted emissions.

Furthermore, the formation of dimers in the solid state was supported by the evidence of fluorescence lifetimes. The dilute solutions of **PyCl**, **PyNO**_**3**_, **PyOTs**, and **PyPh**_**4**_**B** in acetonitrile exhibit very short fluorescence lifetime of ~0.3 ns. However, their fluorescence lifetimes in the solid state are dramatically different and dependent on the species of anions (Fig. 2). The fluorescence lifetime values of **PyCl**, **PyNO**_**3**_, and **PyOTs** were 2.71 ns, 1.85 ns, and 7.81 ns, respectively. By contrast, **PyPh**_**4**_**B** without dimers showed the shortest fluorescence lifetime that is comparable to the value measured in acetonitrile solution. The different fluorescence lifetimes and quantum yields of **PyCl** and **PyNO**_**3**_ in the solid state indicated that the strong interdimeric interactions did not decrease the emission but it would decrease the stability of excited state of dimers.

**Figure 5:**
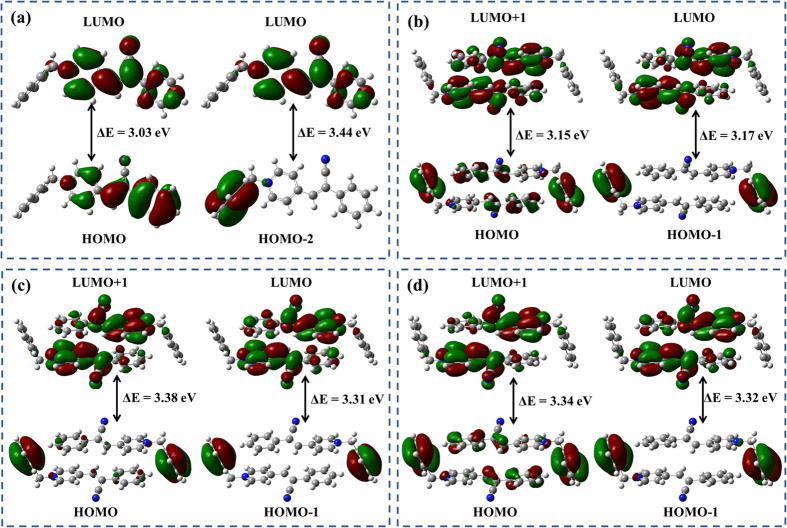
Selected frontier orbitals of the cationic monomer (**a**) and dimers of **PyCl** (**b**), **PyNO**_**3**_ (**c**) and **PyOTs** (**d**) at the singlet states.

The electronic processes of monomer and dimer were also investigated by theoretical calculations. Frontier orbitals energy was illustrated in Table S2. The absorption of the monomer is attributed to transition of HOMO to LUMO and HOMO-2 to LUMO. As shown in Fig. 5, The electronic clouds of HOMO and LUMO located on the cyano substituted styrene unit and have no obviously separation that attributed to the π-π* transition. The HOMO-2 cloud distributes on the phenyl groups. The separation of HOMO-2 and LUMO demonstrates a strong ICT of D-σ-A. In dimers, the benzyl is conjugated through the π bond, which extends the π-conjugation areas. The intradimeric distance of two paralleled molecules conducts the overlap degree of π-conjugation. The intermolecular π-conjugation of **PyCl** are obviously overlap but those of **PyNO**_**3**_ and **PyOTs** are inconspicuous. However, the the strong CT and the appropriate intradimeric distance induce the through-space conjugation and result in the formation of a new lower intradimeric CT state. Furthermore, the fluorescence wavelength of monomer and dimers of **PyCl**, **PyNO**_**3**_ and **PyOTs** were also calculated. The emission of monomer is at 416 nm. The emission wavelengths of dimers were red-shifted compared to monomer and the calculated wavelength of **PyCl**, **PyNO**_**3**_ and **PyOTs** dimers is 472, 437 and 439 nm, respectively. It shows that the dimers contain highly delocalized conjugated system. On the other hand, the calculated wavelength of **PyCl** and **PyOTs** dimer are approximate to the emission wavelength of solid state. But the calculated wavelength of **PyNO**_**3**_ is large blue shifted comparing to the experiments data. It indicated that the interdimeric distance also had effects on the solid emission. The **PyNO**_**3**_ interdimeric distance of is 3.58 Å smaller than that of **PyCl** and **PyOTs**. Thus, it demonstrates that both the interdimeric and intradimeric packing arrangements affect the fluorescence giving rise to the unique solid emission.

**Figure 6:**
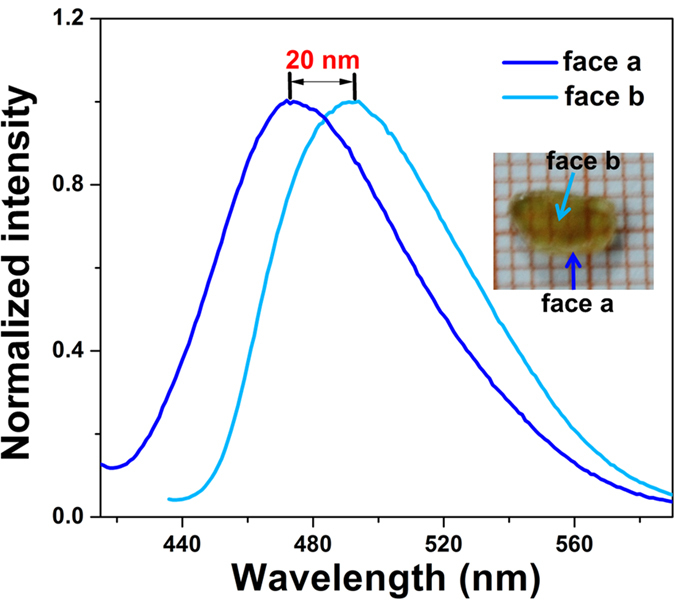
Fluorescence spectra of **PyNO**_**3**_ crystal excited in different directions.

It is interesting that the special arrangement of dimers leads to anisotropic fluorescence of the crystals. It was fortunate that we obtained large volumetric crystals of **PyNO**_**3**_ (5 × 3 × 1 mm), which enable the measurement of anisotropic fluorescence convenient. As shown in Fig. 6, the crystal exhibit fluorescence peaks at 472 nm when it is excited perpendicular to face a. Excitation in the vertical direction of face b induces the emission of dimers, which was red-shifted of 20 nm. Owing to this interesting phenomenon, it may have great potential as anisotropic fluorescent probes for sensing applications.

## Conclusions

In conclusion, we have demonstrated anion-controlled fluorescence of cyanostyryl-based pyridinium and investigated the mechanism at the molecular level. As the key role of anions in molecular packing arrangements, the synergy effects of anions’ steric hindrance and the interactions with cations controlled the monomer-monomer and dimer-dimer distances, which highly affected the solid-state fluorescence. The unique dimeric packing endows the crystal of **PyNO**_**3**_ showing plane anisotropic fluorescence. This anion-controlled approach opens new guideline for the design of highly emissive functional materials.

### Materials and instruments

All reagents were commercially available and used without further purification. All solvents were purified by conventional methods before used. UV-Vis spectra were recorded on a UV-3600 spectrometer with quartz cuvettes of 1.0 cm path length. The fluorescence spectra were recorded with a Hitachi F-7000 Fluorescence spectrometer. Fluorescence lifetime measurements were carried out using an HORIBA FluoroMax-4P fluorescence spectrometer equipped with a time-correlated single-photon counting (TCSPC) card. Absolute fluorescence quantum yields of solid state were determined by a calibrated integrating sphere. FT-IR spectra were obtained in KBr discs on a Nicolet 380 FT-IR spectrometer in the region of 4000–400 cm^−1^. ^1^H NMR and ^13^C NMR were recorded on a Bruker Avance 400 MHz NMR spectrometer using CDCl_3_ or DMSO-d_6_ as solvent. Both ^1^H and ^13^C NMR were used tetramethylsilane (SiMe_4_) as internal standards. NMR data were reported as follows: chemical shifts (*δ*) in ppm, multiplicity (s = singlet, d = doublet, t = triplet, m = multiplet), coupling constants *J* (Hz), integration, and interpretation. Mass spectra were recorded on an Agilent 6410 LC-MS/MS system (USA) equipped with an electrospray ion source (ESI). Silica gel 60 (200–300 mesh) was used for column chromatography. The time-dependent density functional theory (TD-DFT) implemented at the b3lyp/6–31 g* level using the Gaussian 09 program^59^,^60^,^61^,^62^. To improve calculation accuracy, the calculations were performed based on the crystal structure.

### Synthesis of 2-(4-phenyl)-3-(4-pyridinyl)acrylonitrile

A mixture of 4-pyridinecarboxaldehyde 1.07 g (10 mmol) and phenylacetonitrile 1.17 g (10 mmol) in water (50 mL) was stirred at room temperature. Then, 8 mL 5% aqueous solution of NaOH was added by dropwise into the flask over 1 h. The mixture was sequentially stirred for another 12 h. The precipitate was filtered and washed with water. 1.94 g white solid was obtained, yielded 94%. ^1^H NMR (DMSO-d_6_, 400 MHz, ppm) *δ*: 8.77 (d, *J* = 5.6 Hz, 2 H), 8.11 (s, 1 H), 7.82 (d, *J* = 6.4 Hz, 4 H), 7.50–7.58 (m, 3 H). ^13^C NMR (DMSO-d_6_, 100 MHz, ppm) *δ*: 114.70, 116.97, 122.62, 126.13, 129.27, 130.07, 132.93, 140.20, 140.74, 150.43. HR-MS (ESI-MS): m/z = 207.0918, calcd for (C_14_H_11_N_2_)^+^ = 207.0922 ([M+H]^+^).

### Synthesis of compound PyCl

2-(4-phenyl)-3-(4-pyridinyl)acrylonitrile (0.206 g, 1 mmol) was added to 5 mL benzyl chloride. The reaction was heated to reflux for overnight. After cooling down to room temperature, the mixture was filtered and the solid was washed with THF and toluene. The solid was dried under reduced pressure to give yellow wish solid 0.281 g, yielded 94%. FT-IR (KBr, cm^−1^): 3423 (w), 3124 (w), 3027(s), 2968 (m), 2222 (w), 1637 (vs), 1603 (s), 1593 (m), 1556 (m), 1522 (m), 1496 (m), 1469 (m), 1454 (m), 1367 (w), 1350 (w), 1280 (w), 1261 (w), 1218 (w), 1208 (w), 1156 (s), 1077 (w), 1033 (w), 1003 (w), 932 (w), 920 (w), 853 (w), 815 (m), 767 (s), 742 (s), 705 (m), 686 (m), 671 (m), 611 (m), 550 (w), 523 (m), 452 (w). ^1^H NMR (DMSO-d_6_, 400 MHz, ppm) *δ*: 5.92 (s, 2 H), 7.47 (d, 3 H), 7.60 (t, 5 H), 7.89 (t, 2 H), 8.39 (s, 1 H), 8.51 (d, 2 H), 9.36 (d, 2 H). ^13^C NMR (DMSO-d_6_, 100 MHz, ppm) *δ*: 63.00, 116.15, 119.61, 126.74, 127.10, 128.97, 129.24, 129.43, 129.51, 131.31, 132.18, 134.20, 136.40, 145.22. HR-MS (ESI-MS): m/z = 297.1477, calcd for (C_21_H_17_N_2_)^+^ = 297.1392 ([M-Cl]^+^).

### Synthesis of compound PyNO_3_

**PyCl** (0.166 g, 0.5 mmol) was dissolved in 5 mL MeOH and equimolar AgNO_3_ was added. The mixture was vigorously stirred at room temperature for 3 h. then, the precipitate was filtered. The MeOH was evaporated under vacuum. Giving **PyNO**_**3**_ 0.167 g, yielded 93%.

### Synthesis of silver p-toluenesulfonate

To 30 mL Na_2_CO_3_ (5.3 g, 0.05 mol) aqueous solution, 30 mL AgNO_3_ (17 g, 0.1 mol) aqueous solution was added. The precipitate of Ag_2_CO_3_ was collected by filtration and washed with water. Then, Ag_2_CO_3_ was added to 200 mL water. To the suspension of Ag_2_CO_3_, p-toluenesulfonic acid monohydrate (19 g, 0.1 mol) was added. After stirring for 30 mins, a clear solution was obtained by filtration. The water was evaporated slowly on a hotplate to give 25.9 g white solid, yielded 93%.

### Synthesis of compound PyOTs

**PyOTs** was prepared in a similar method to **PyNO**_**3**_.

### Synthesis of compound PyPh_4_B

**PyCl** (0.66 g, 2.0 mmol) was dissolved in 10 mL MeOH, and then, 0.85 g tetraphenylborate in 10 mL EtOH was added. The mixture was stirred at room temperature for 10 mins. The precipitate was filtered and washed with water and MeOH to give **PyPh**_**4**_**B** 0.92 g, yielded 74%.

## Additional Information

**How to cite this article**: Zhang, G. *et al*. Anion-controlled dimer distance induced unique solid-state fluorescence of cyano substituted styrene pyridinium. *Sci. Rep.* **6**, 37609; doi: 10.1038/srep37609 (2016).

**Publisher’s note**: Springer Nature remains neutral with regard to jurisdictional claims in published maps and institutional affiliations.

## Supplementary Material

Supplementary Information

## Figures and Tables

**Figure 1:**
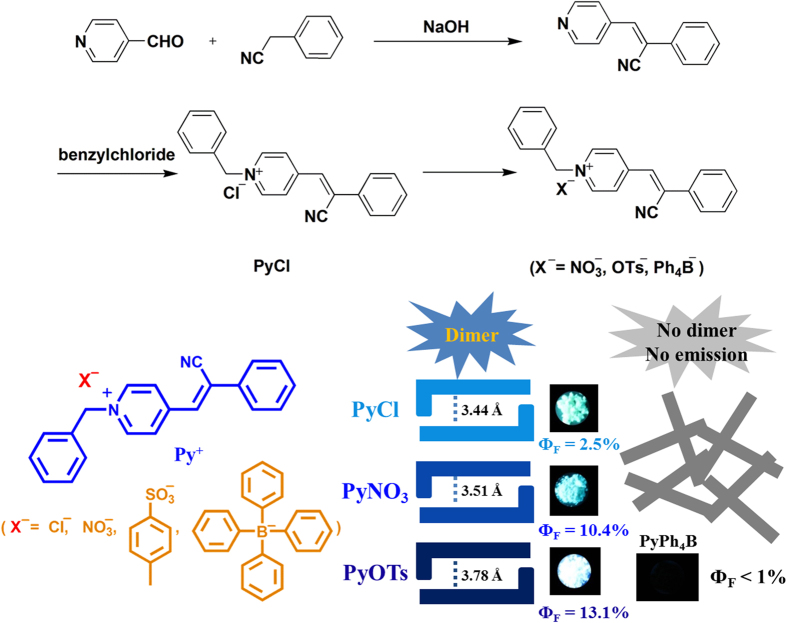
The synthetic routes of compounds and schematic illustration of packing arrangements.

**Table 1:** Photophysical properties of the compounds.

Compounds	λ_em_^a^ (nm)	Ф_F_^b^ (%)	Ф_F_^c^ (%)	τ^e^ (ns)	τ^f^ (ns)
**PyCl**	486	2.5	0.2	0.14	2.71
**PyNO**_**3**_	467	10.4	0.3	0.26	1.85
**PyOTs**	448	13.1	0.1	0.16	7.81
**PyPh**_**4**_**B**	—	<1	—	0.11	0.1

^a^Emission wavelength in the solid state.

^b^Absolute fluorescence quantum yields of solids determined by a calibrated integrating sphere.

^c^Absolute fluorescence quantum yields in acetonitrile of compounds determined by a calibrated integrating sphere.

^d^Fluorescence lifetimes in acetonitrile.

^e^Fluorescence lifetimes of the solid samples.
