# Supplementary material for: Anion-controlled dimer distance induced unique solid-state fluorescence of cyano substituted styrene pyridinium
Source: Sci Rep. 2016 Nov 21;6:37609. doi: 10.1038/srep37609 (PMC5116621; doi:10.1038/srep37609)
Supplement: Supplementary Information [file srep37609-s1.pdf]

Supporting Information

Anion-controlled dimer distance induced unique solid-state fluorescence of cyano substituted styrene pyridinium

Gaobin Zhang,[a] Xuanjun Zhang,*[b] Lin Kong,[a] Shichao Wang,[b] Yupeng Tian,[a] Xutang Tao,[c] and Jiaxiang Yang*[ac]

[a]*College of Chemistry & Chemical Engineering, Key Laboratory of Functional Inorganic Materials of Anhui Province, Anhui University, Hefei 230039, P. R. China;* [b]*Faculty of Health Sciences, University of Macau, Taipa, Macau SAR, P. R. China,* [c]*State Key Laboratory of Crystal Materials, Shandong University, Jinan 502100, P. R. China.*

**Corresponding authors.* Tel: (+853) 8822 4928; (+86) 551-63861279; E-mail address: xuanjunzhang@umac.mo; jxyang@ahu.edu.cn

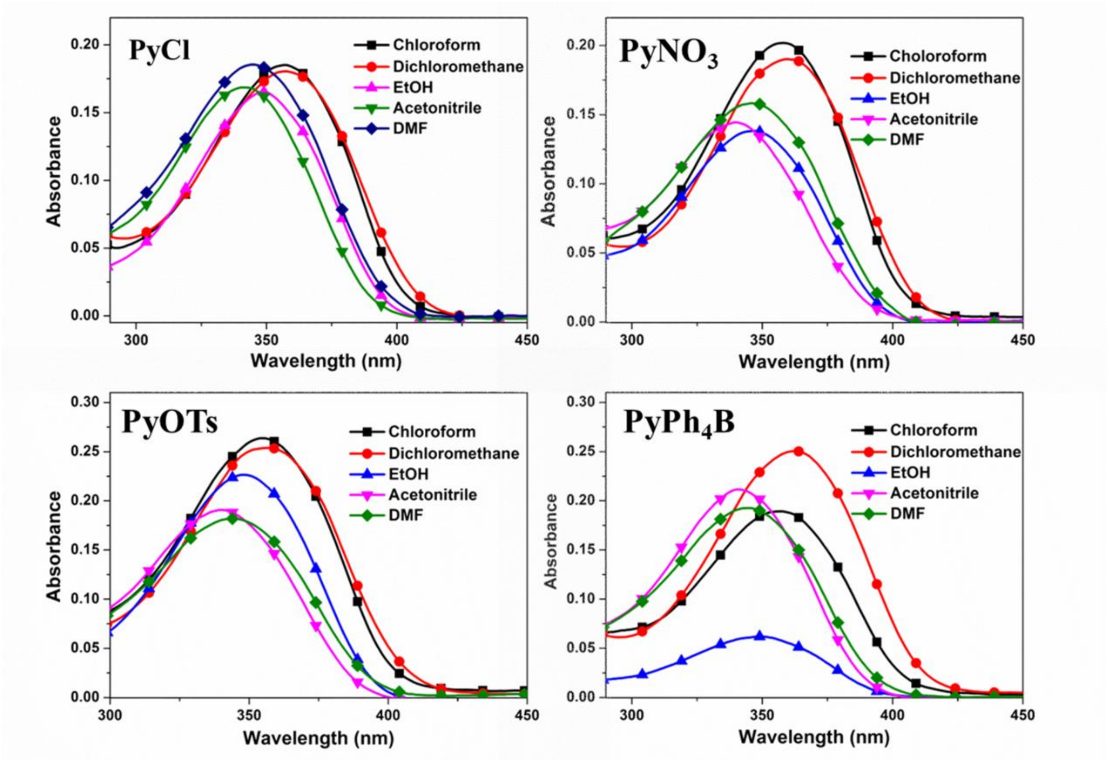

Figure S1. The absorption spectra of **PyCl**, **$PyNO_3$**, **PyOTs** and **$PyPh_4B$** in solution (Chloroform, Dichloromethane, EtOH, Acetonitrile, DMF; 1× 10^{-5} M)

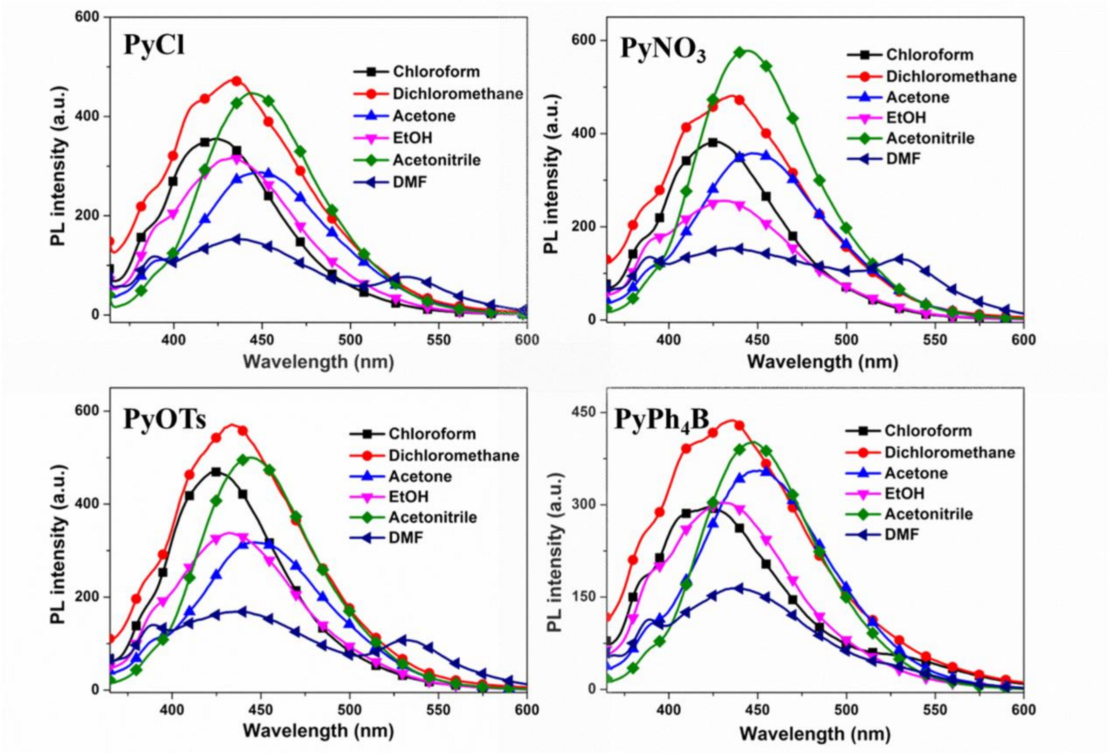

Figure S2. The fluorescence spectra of **PyCl**, **$PyNO_3$**, **PyOTs** and **$PyPh_4B$** in solution (Chloroform, Dichloromethane, EtOH, Acetonitrile, DMF; 1× 10^{-5} M)

Lippert-Mataga equation

The effect of solvent on the optical features of compounds can be further evaluated by Lippert-Mataga equation (eqs 1 and 2),[2] which describes the relationship between the solvent polarity parameter (Δf) and Stokes' shift of the absorption and emission maxima. Where $\Delta \upsilon$ is the Stokes' shift, h is the Planck constant, c is the speed of light, a is the Onsager cavity radius, μ_e is the excited state dipole moment, μ_g is the ground-state dipole moment; ε and n are the solvent dielectric and the solvent refractive index.

$$\Delta \nu = \nu_{ab}\text{-}\nu_{em} = \frac{2\Delta f}{hca^3}(\mu_e - \mu_g)^2 + \text{constant} \qquad (1)$$

$$\Delta f = f(\varepsilon) - f(n^2) \approx \frac{\varepsilon - 1}{2\varepsilon + 1} - \frac{n^2 - 1}{2n^2 + 1} \qquad (2)$$

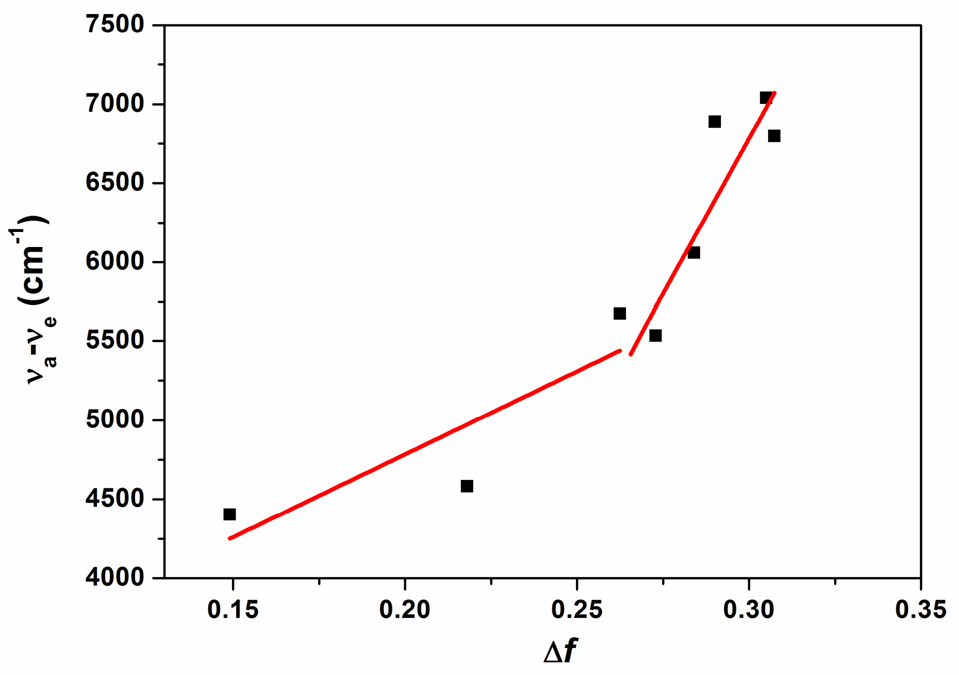

Figure S3. Linear correlation of the Stokes shift with the solvents orientation polarization for **PyNO3**

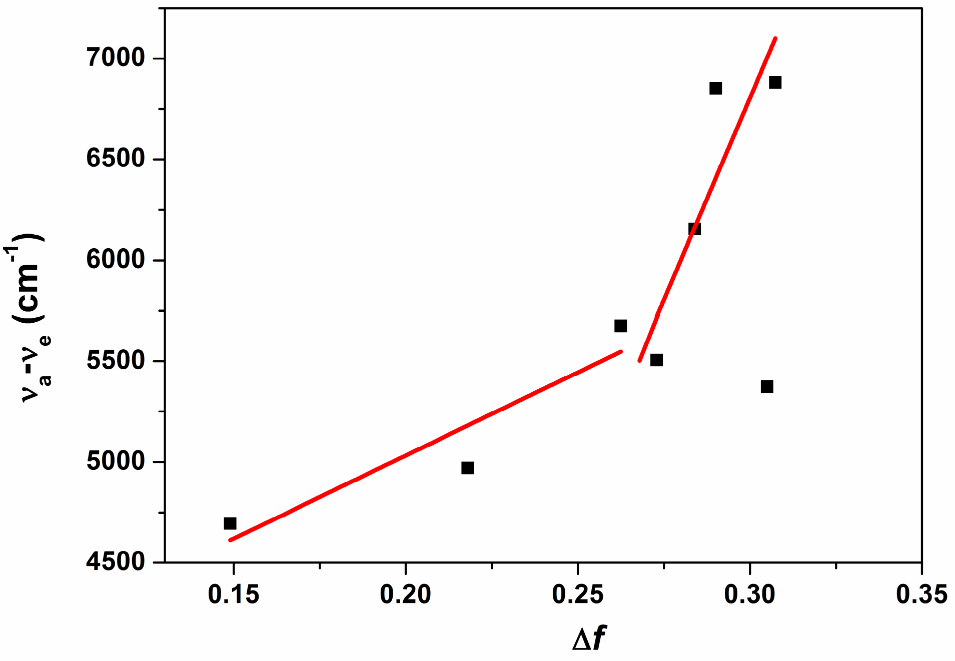

Figure S4. Linear correlation of the Stokes shift with the solvents orientation polarization for **PyOTs**

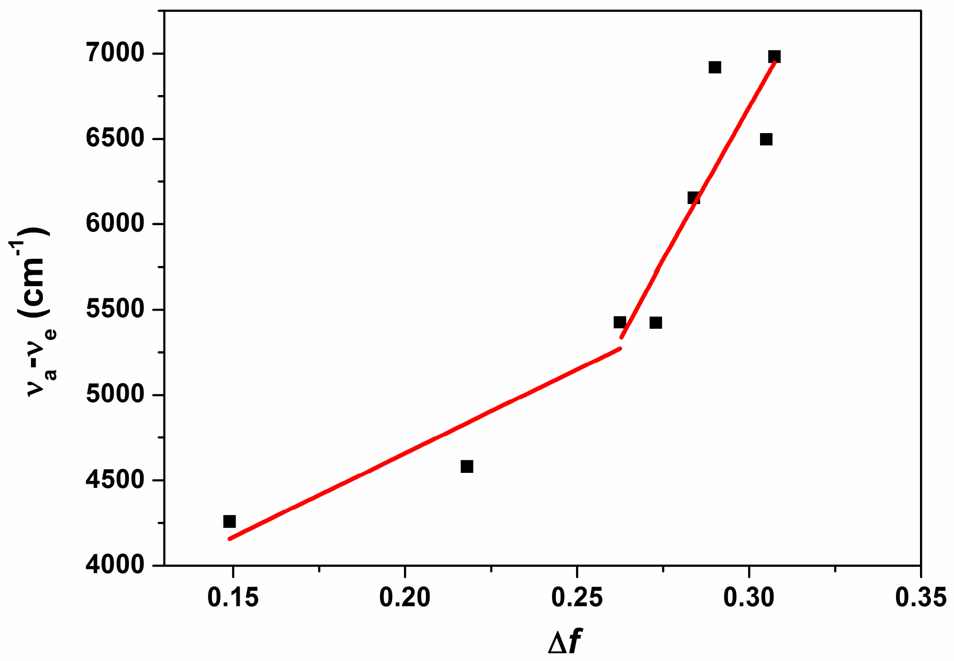

Figure S5. Linear correlation of the Stokes shift with the solvents orientation polarization for

$\mathbf{PyPh_4B}$

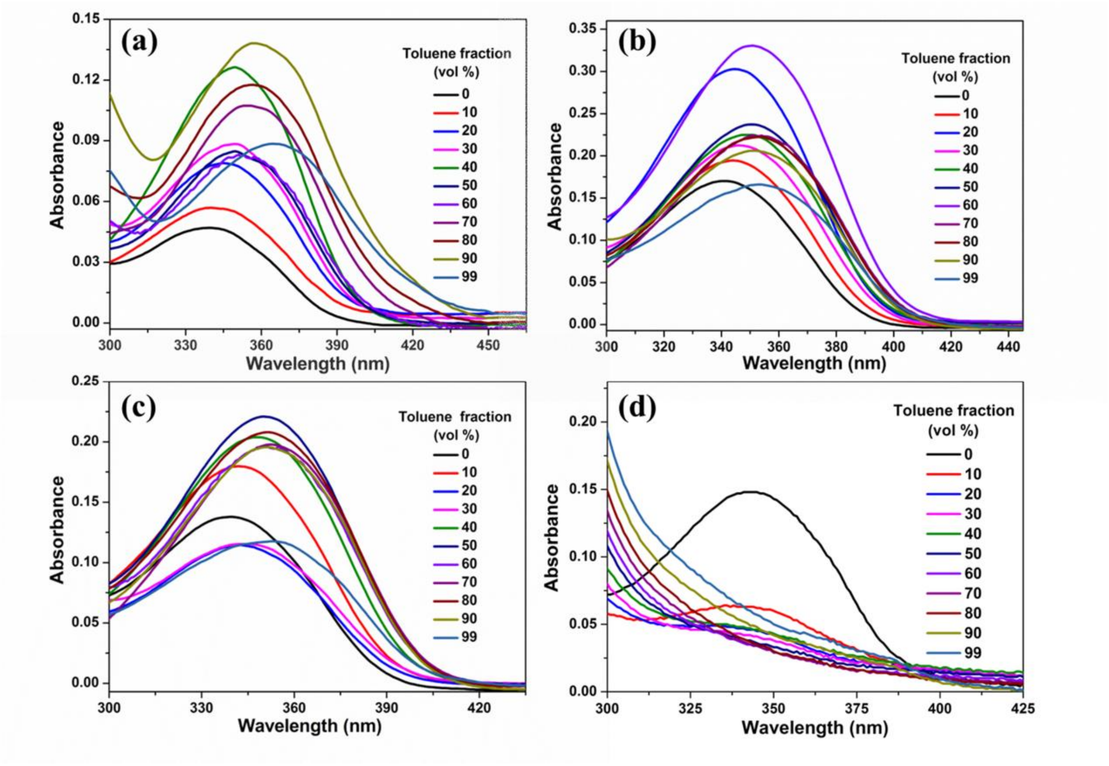

Figure S6. Absorption spectra of **PyCl** (a), **$PyNO_3$** (b), **PyOTs** (c) and **$PyPh_4B$** (d) in acetonitrile/toluene mixture solution with different toluene fractions (1×10^{-5} M)

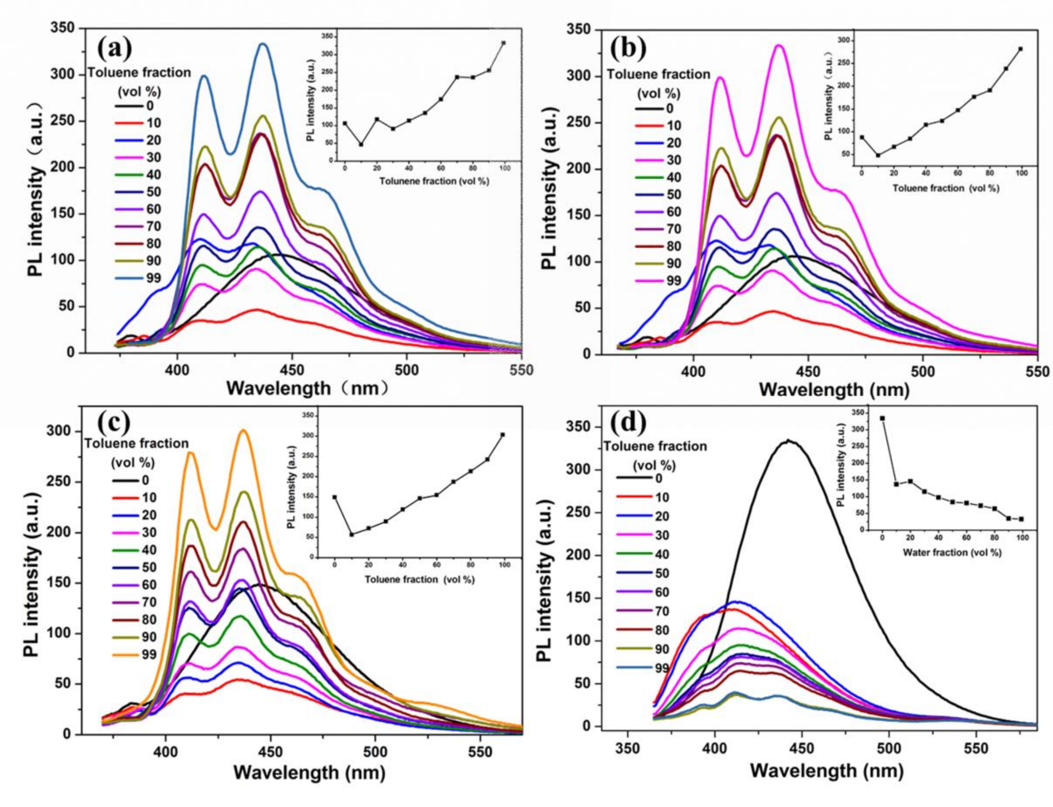

Figure S7. Fluorescence spectra of **PyCl** (a), **$PyNO_3$** (b), **PyOTs** (c) and **$PyPh_4B$** (d) in acetonitrile/toluene mixture solution with different toluene fractions (1×10^{-5} M)

3. Crystallography

Single-crystal X-ray diffraction measurements were collected on a Siemens Smart 1000 CCD diffractometer using graphite amonochromated Mo Kα radiation (λ = 0.71069 Å) at 296(2) K to determinate unit cell parameters. Intensity data were collected in the variable ω-scan mode. The structures were solved by direct methods and refined on F2 by using full-matrix least-squares methods with the SHELXL-97 program package. All non-hydrogen atoms were located in successive difference Fourier syntheses and refined with anisotropic thermal factors. The hydrogen atoms were introduced geometrically. CCDC 1485583 (**PyCl**), 1485459 (**PyNO$_3$**), 1485584 (**PyOTs**) and 1485422 (**PyPh$_4$B**) contains the supplementary crystallographic data for this paper. The data can be obtained free of charge from The Cambridge Crystallographic Data Centre via www.ccdc.cam.ac.uk/structures.

Table S1. The detailed crystal and structure refinement data of the compounds

Compound	**PyCl**	**PyNO$_3$**	**PyOTs**	**PyPh$_4$B**
empirical formula	$C_{21}H_{17}ClN_2$	$C_{21}H_{17}N_3O_3$	$C_{28}H_{24}N_2O_3S$	C_{90} H_{74} B_2 N_4
formula weight	332.82	359.38	468.55	1233.15
crystal system	Monoclinic	Monoclinic	Monoclinic	Orthorhombic
space group	P2(1)/c	P2(1)/c	P2(1)/c	Pna2(1)
a [Å]	8.346(4)	8.8381(17)	11.344(5)	21.777(8)
b [Å]	9.464(5)	9.5668(19)	23.131(5)	10.675(3)
c [Å]	22.652	22.090(4)	10.419(5)	30.125(10)
α [°]	90	90	90	90
β [°]	94.934(8)	93.778(2)	114.529(5)	90
γ [°]	90	90	90	90
V [Å^3]	1782.7(16)	1863.7(6)	2487.2(17)	7003(4)
Z	4	4	4	4
T [K]	296(2)	296(2)	296(2)	296(2)
D called [g·cm^{-3}]	1.240	1.281	1.251	1.170
M [mm^{-1}]	0.217	0.088	0.162	0.067
θ rang [°]	1.80-24.99	1.85- 25.00	1.76-25.00	1.35-25.00
total no. data	8685	12852	12737	47923
no. unique data	3118	3291	4379	12307
no. params refined	217	244	308	866
R_1	0.1166	0. 0605	0.0439	0.0714
wR_2	0.3385	0.1819	0.1547	0.1601
GOF	1.006	0.977	0.996	0.930

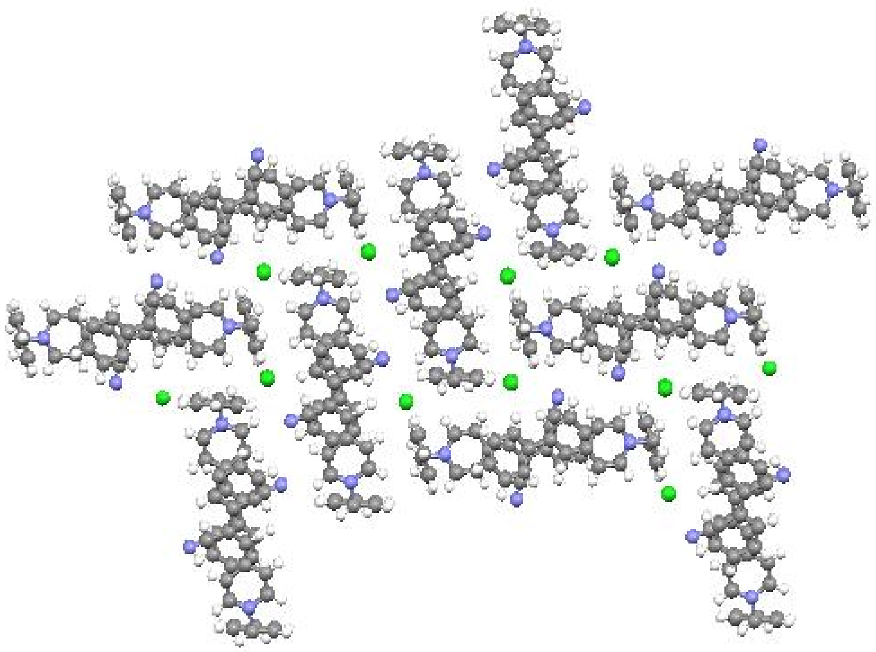

Figure S8. The molecular packing arrangements of **PyCl**

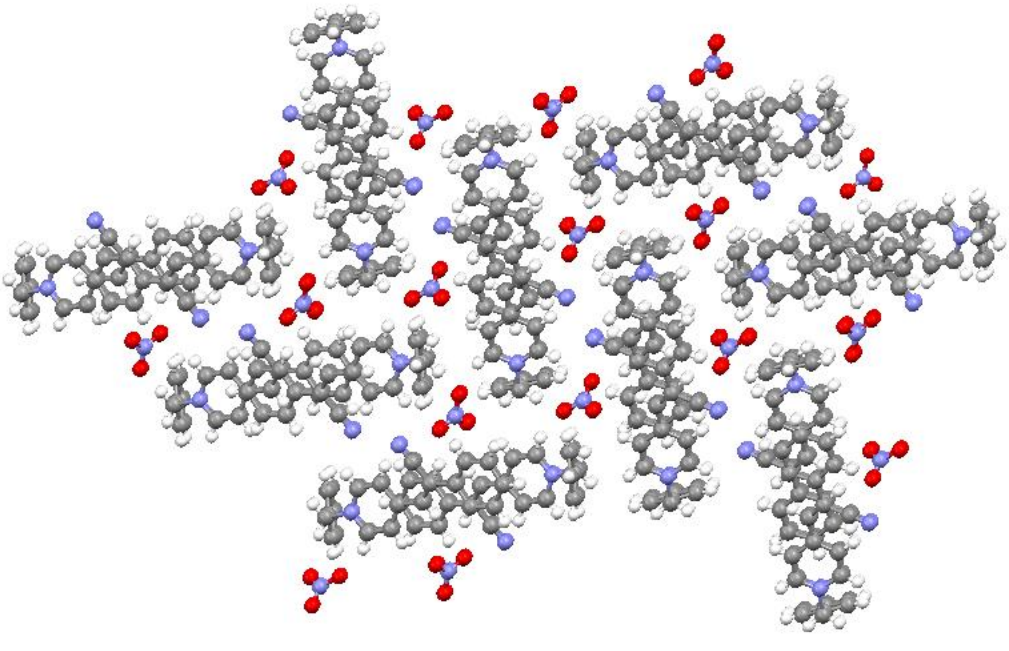

Figure S9. The molecular packing arrangements of **$PyNO_3$**

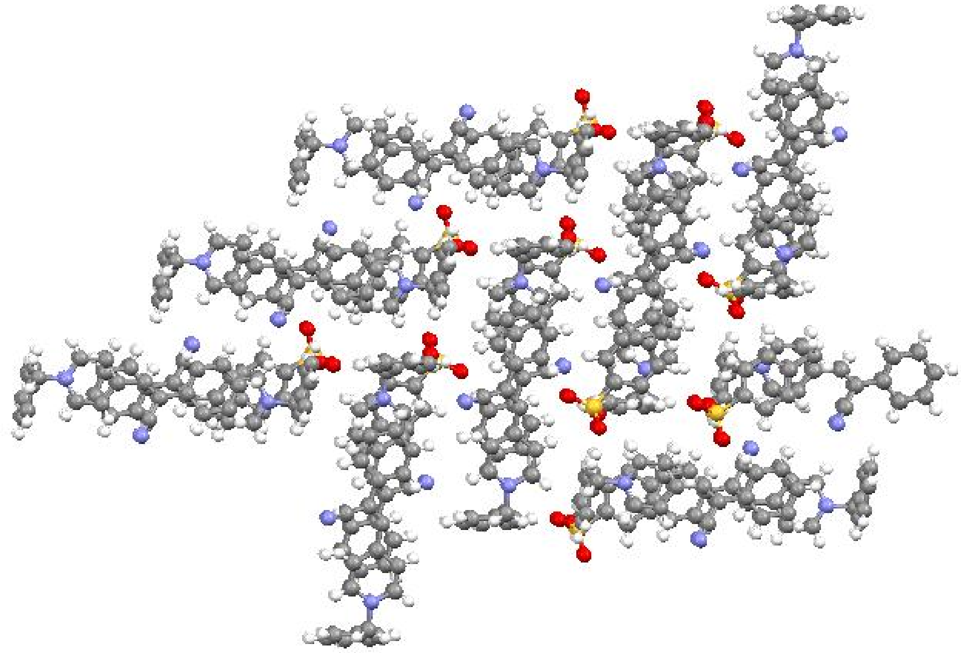

Figure S10. The molecular packing arrangements of **PyOTs**

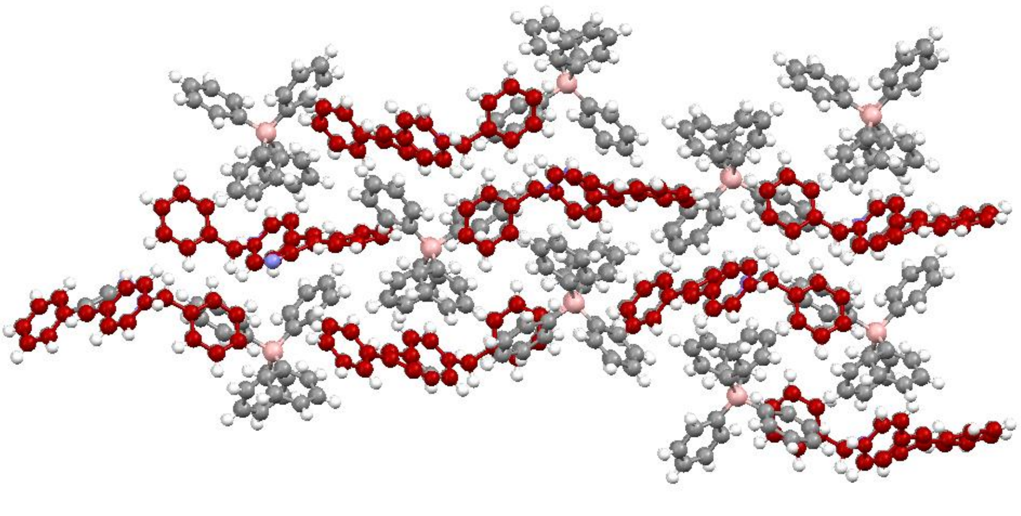

Figure S11. The molecular packing arrangements of **PyPh$_4$B**

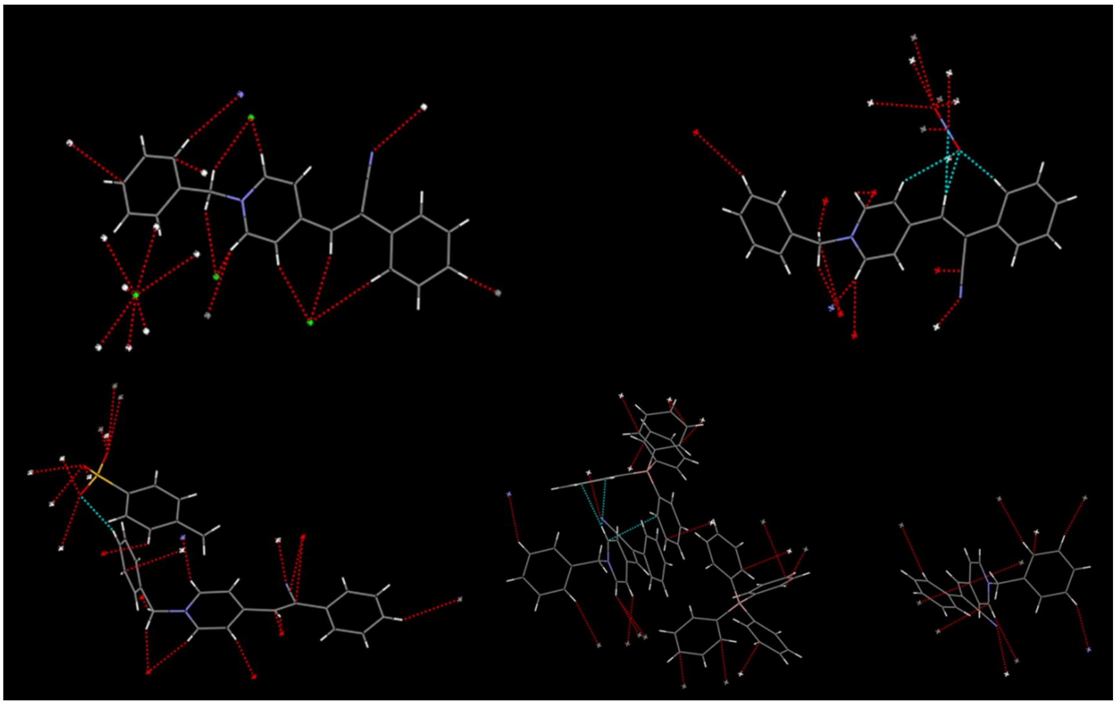

Figure S12. Crystal structures of the compounds with denoted intermolecular interactions

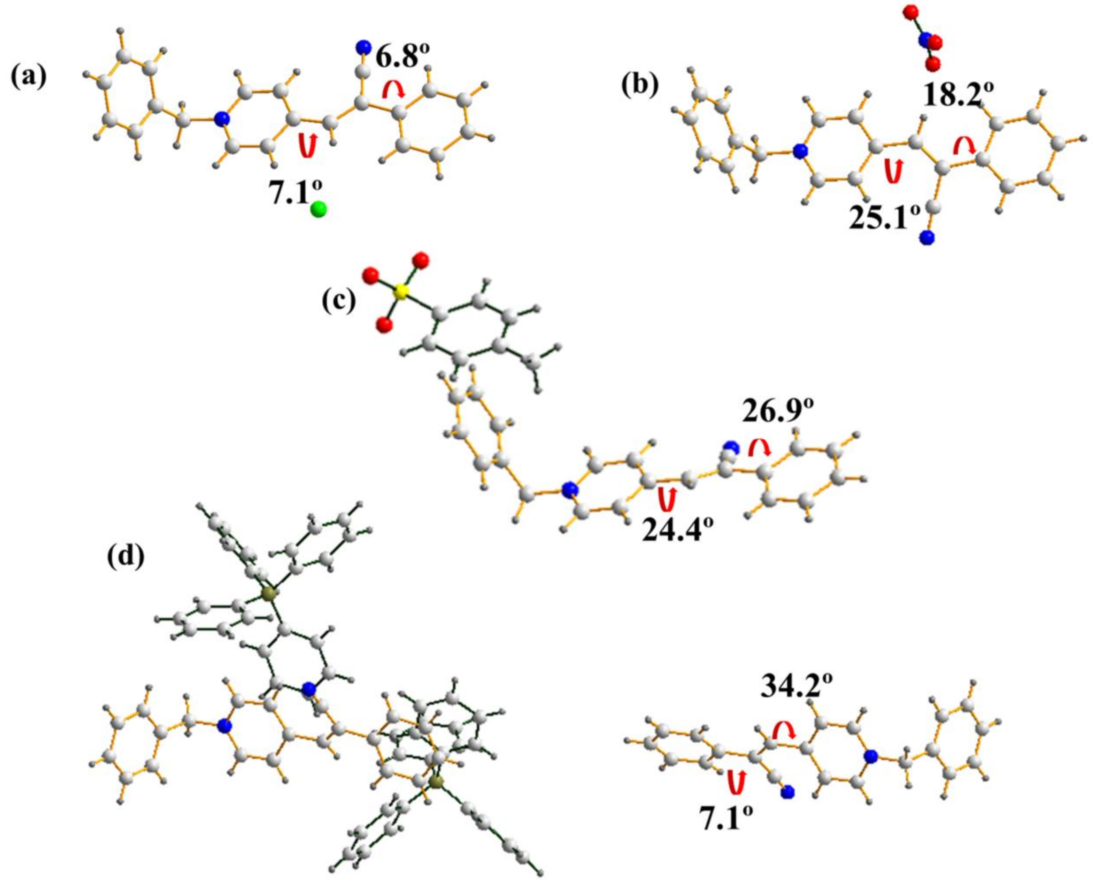

Figure S13. Crystal structures and the dihedral angles of **PyCl** (a), **PyNO$_3$** (b), **PyOTs** (c) and **PyPh$_4$B** (d).

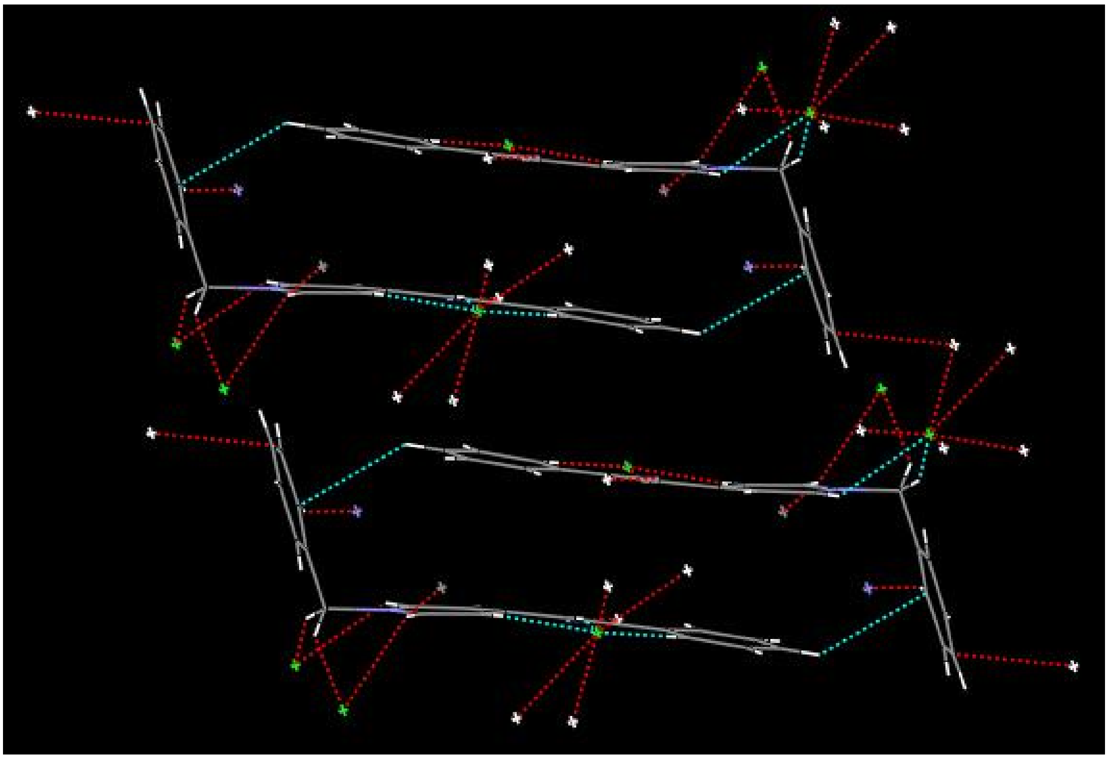

Figure S14. Partial molecular arrangement of **PyCl** cocrystal with denoted intermolecular interactions

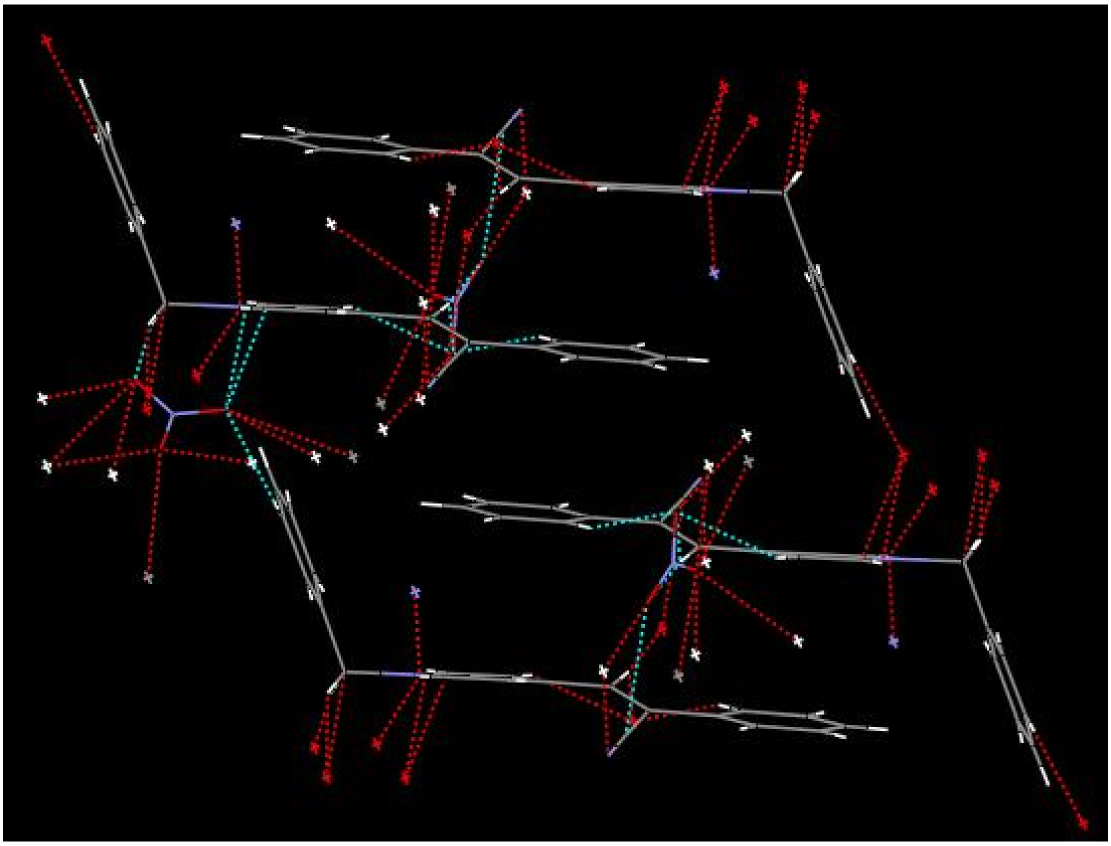

Figure S15. Partial molecular arrangement of **PyNO$_3$** cocrystal with denoted intermolecular interactions

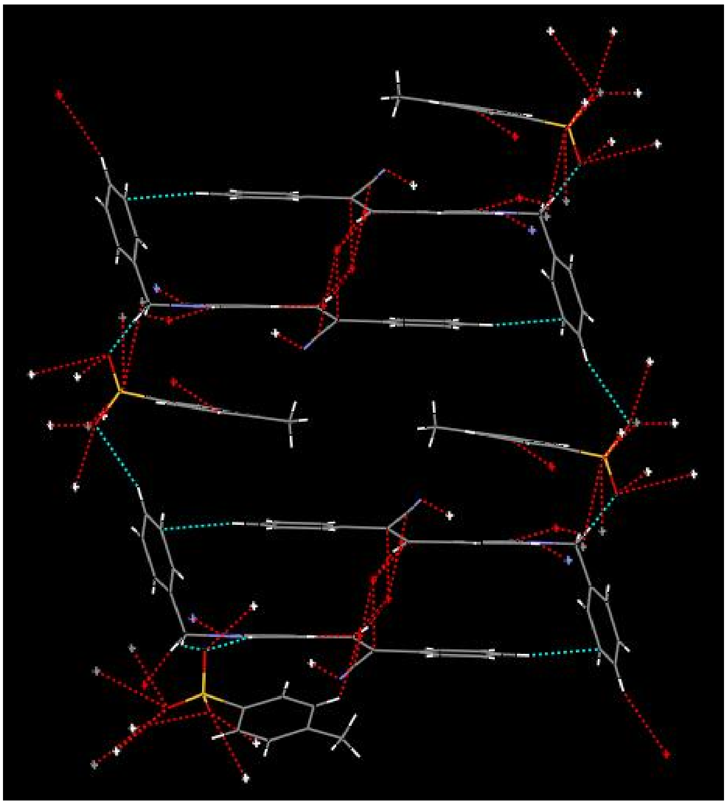

Figure S16. Partial molecular arrangement of **PyOTs** cocrystal with denoted intermolecular interactions

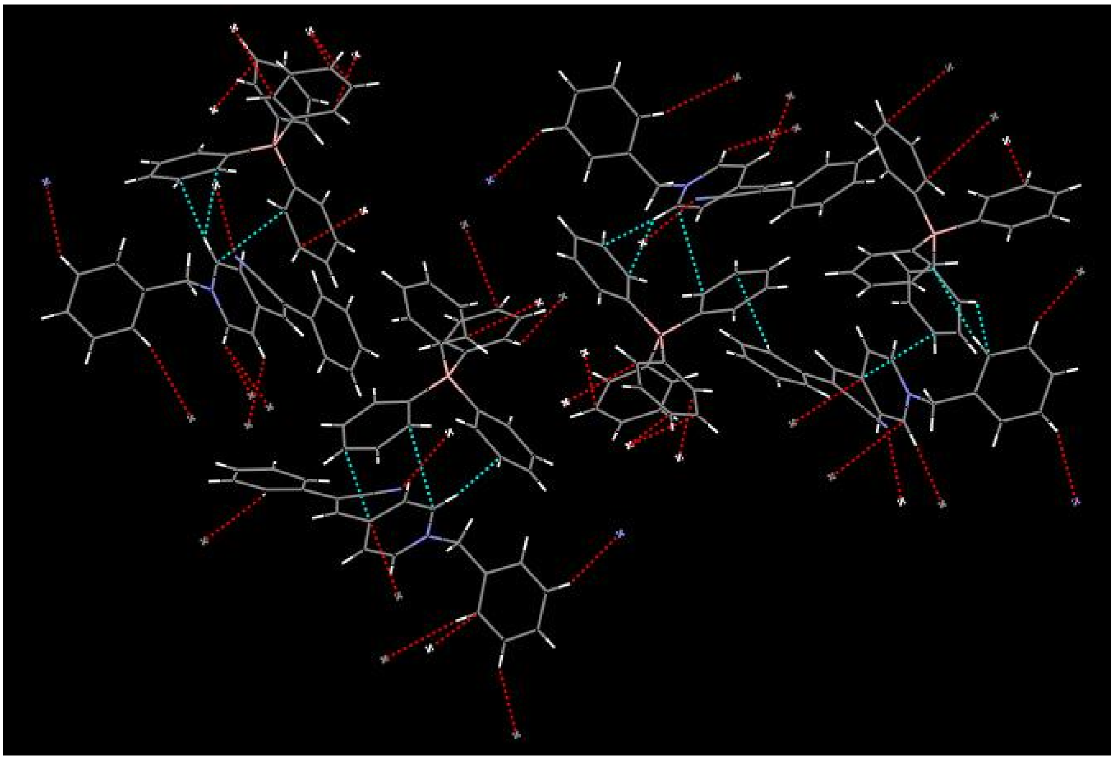

Figure S17. Partial molecular arrangement of **PyPh$_4$B** cocrystal with denoted intermolecular interactions

Table S2. The molecular orbital energy of the compounds

Compounds	LUMO+1 (eV)	LUMO (eV)	HOMO (eV)	HOMO-1 (eV)
**monomer**	-4.74	-6.42	-9.45	-9.79
**PyCl dimer**	-8.33	-8.34	-11.48	-11.51
**$PyNO_3$ dimer**	-8.19	-8.27	-11.57	-11.58
**PyOTs dimer**	-8.18	-8.24	-11.53	-11.57

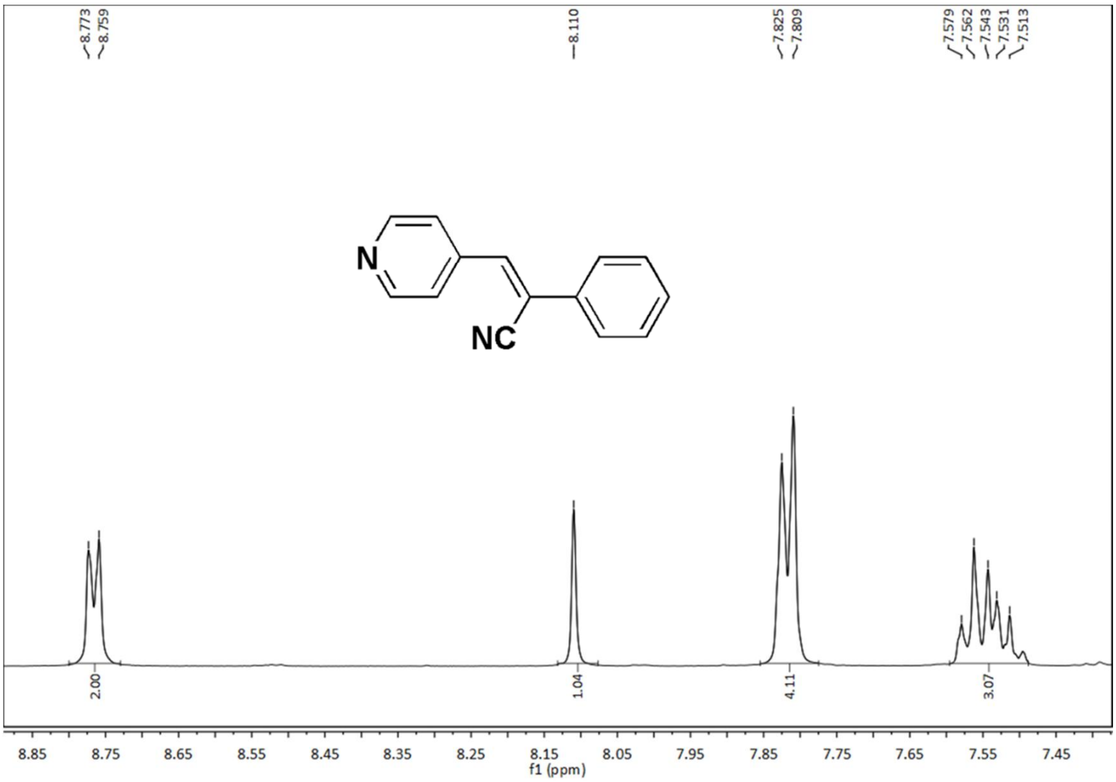

Figure S18. The ^{1}H NMR of 2-(4-phenyl)-3-(4-pyridinyl)acrylonitrile

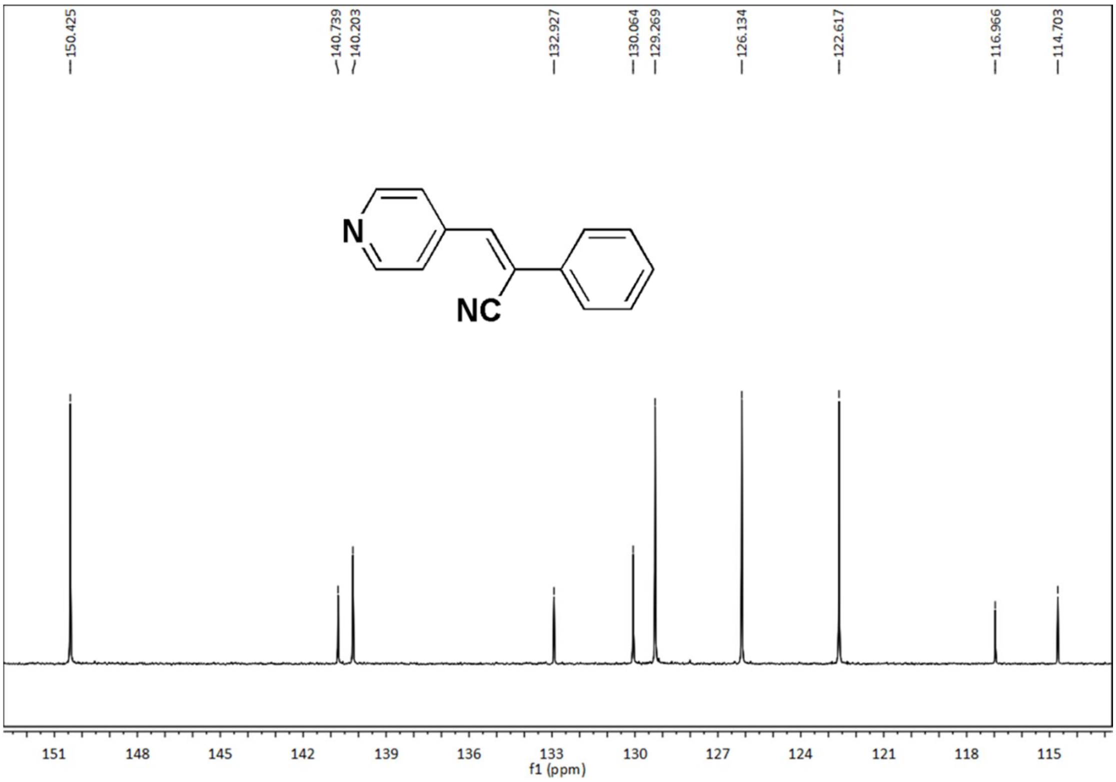

Figure S19. The ^{13}C NMR of 2-(4-phenyl)-3-(4-pyridinyl)acrylonitrile

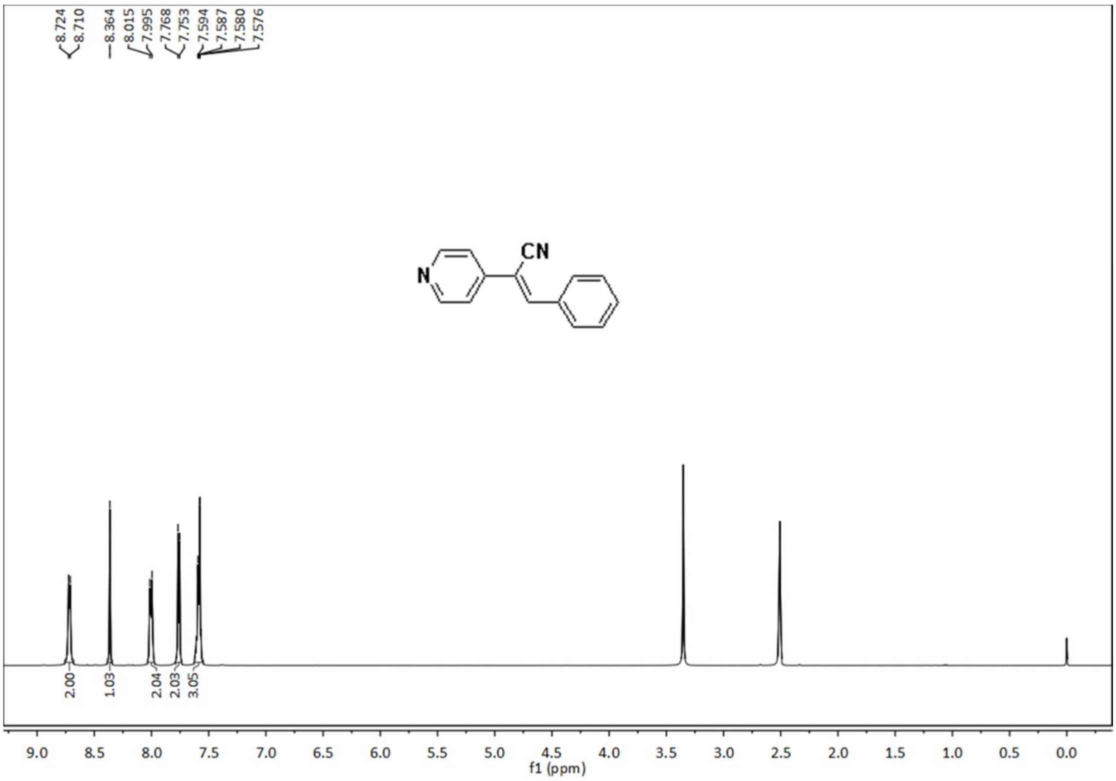

Figure S20. The ^{1}H NMR of 2-(4-pyridinyl)-3-(4-phenyl)acrylonitrile

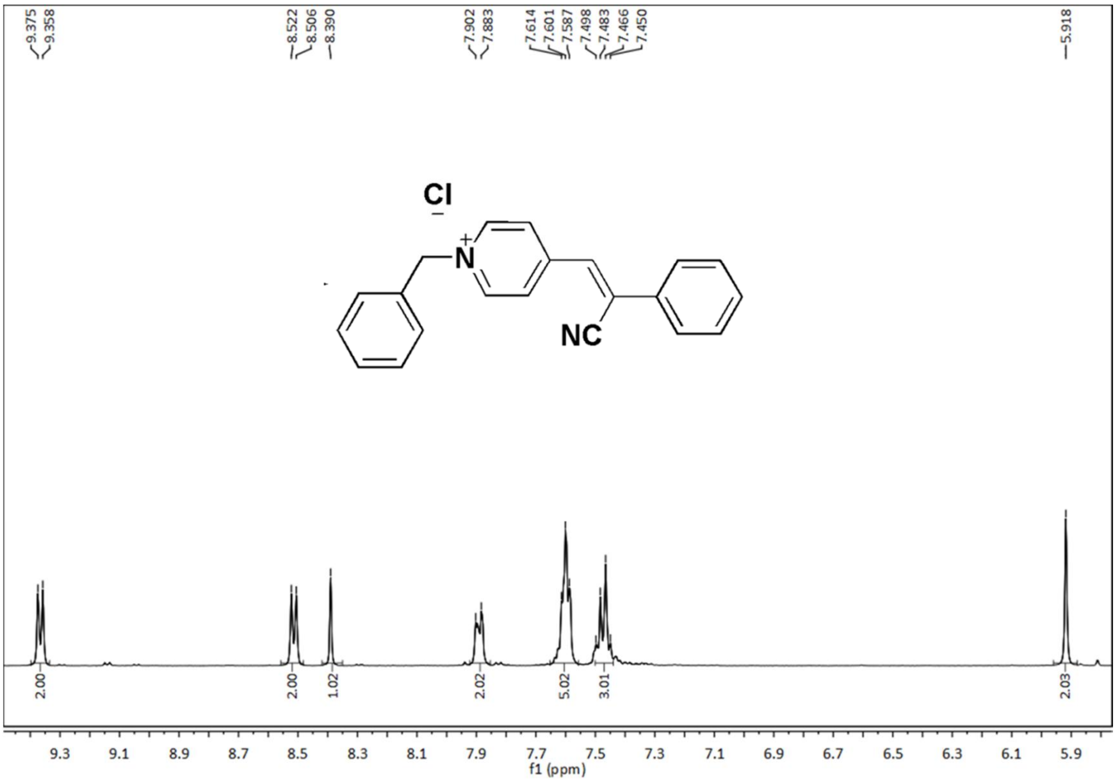

Figure S21. The ^{1}H NMR of **PyCl**

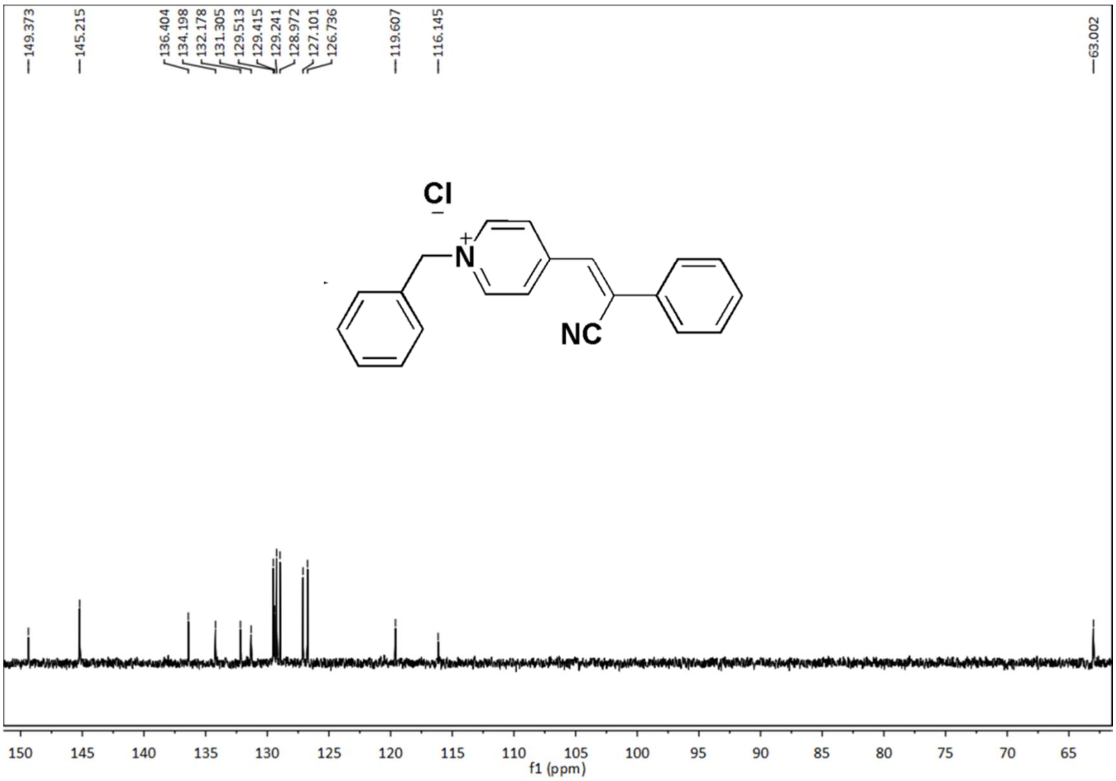

Figure S22. The ^{13}C NMR of **PyCl**
